# The collateral damage of the COVID-19 pandemic on homeless people in the Netherlands; a qualitative study on the impact of health and care

**DOI:** 10.3389/fmed.2024.1305834

**Published:** 2024-03-07

**Authors:** Tessa van Loenen, Jeyna Sow, Maria van den Muijsenbergh

**Affiliations:** ^1^Department of Primary and Community Care, Radboud University Medical Center, Nijmegen, Netherlands; ^2^Pharos, Centre of Expertise on Health Disparities, Utrecht, Netherlands

**Keywords:** homeless, COVID-19, mental health, primary care, qualitative

## Abstract

**Introduction:**

People experiencing homelessness, also in the Netherlands, experience poorer physical and mental health compared to the general population and suffer from unmet health needs that are strongly related to their unfavorable social situation. This makes them especially vulnerable to negative consequences of a public health emergency such as the COVID-19 pandemic. This qualitative study aims to provide insight into the experiences of people experiencing homelessness with the impact of the pandemic on their health and lives.

**Methods:**

We performed semistructured interviews at 3 different times in the first 2 years of the pandemic including, respectively, 67, 55, and 53 persons. Interviews focused on their experienced mental and physical health, their experiences with the public health measures taken, and the care they received during the pandemic.

**Results:**

In each round of interviews, the self-reported mental health was lower than before. In the last round approximately half felt mentally unhealthy. Mental health was negatively impacted due to livelihood insecurity, loss of social contact and poor accessibility to social and medical care. Twenty-four hour shelter locations with smaller dormitories had a positive impact on mental health.

**Conclusion and recommendations:**

Most preventive measures taken during the pandemic negatively impacted the mental health of people experiencing homelessness but some improved their health. We recommend special attention to the effects on mental health when planning measures for pandemic control and we recommend to implement 24-h shelter and smaller dormitories.

## 1 Introduction

On March 11th, 2020, the World Health Organization (WHO) declared COVID-19 as a pandemic. While everyone is at risk of contracting the virus, individuals with underlying health conditions or poor general health are at higher risk for severe outcomes (1). This is particularly relevant for people experiencing homelessness who suffer from worse health than the general population and are more susceptible to infections due to crowded shelters and poor hygiene facilities (2–4). In 2020, the Netherlands counted approximately 36,000 persons experiencing homelessness (5). However, the actual number may deviate from the actual figures since this number is based on estimates.

During the pandemic, governments implemented strict public health and social measures (PHSM) to prevent the spread of the virus and reduce mortality and morbidity, such as hygiene measures, facemasks, physical distancing, and the closure of schools, workplaces and sometimes face-to-face healthcare (6–8). However, these policies had a significant impact on daily life and caused economic and social disruptions. Recent studies show that the ongoing pandemic and the measures to contain the virus have taken a toll on the mental health of many people, particularly those in vulnerable positions resulting in a widening socioeconomic health gap (1, 9–11).

People experiencing homelessness are particularly vulnerable to the economic and health consequences of the pandemic, as they live in extreme socioeconomic vulnerability and have limited opportunities to comply with COVID-19 preventive measures such as physical distancing or staying at home (12, 13). The wellbeing of this group is intricately tied to their social circumstances, notably financial strain and feelings of isolation (4). Even before the onset of the COVID-19 pandemic, persons experiencing homelessness in the Netherlands were already grappling with poor physical and mental health. Research conducted by Verheul revealed that 19% of the surveyed population rated their self-reported health as either poor or very poor (4). Furthermore, a significant majority (57%) expressed experiencing feelings of sadness.

The COVID-19 response for the population of people experiencing homelessness brought about extended operating hours of shelters, and the creation of smaller dormitories. However, it also resulted in the closure of daytime activities and programs. In the Netherlands, pre-COVID, government support for this group varied based on entitlement. Those entitled had access to shelter, food and hygiene with efforts to secure permanent housing. Conversely, those not entitled such as undocumented migrants, had limited access to support, often relying on inconsistent charitable aid. During lockdowns, those not entitled in usual circumstances gained for short periods access to shelter. All these groups were entitled to access healthcare, although undocumented people are not allowed to have health insurance.

Some global studies have illustrated how the pandemic has affected this population, encompassing health, social, and economic dimensions (14–17). A study in the Netherlands showed that the actual COVID-19 infection among individuals experiencing homelessness was less than previously anticipated, and contradictory to some other countries (12, 18–21). Limited information exists about how these people have coped with PHSM and pandemic-related life disruptions.

### 1.1 Research aim

This study aims to shed light on the experiences of persons experiencing homelessness in the Netherlands during the pandemic and the effects they experienced on their mental wellbeing and lives. Understanding these factors can offer valuable insights for molding future pandemic responses and formulating interventions that tackle the lasting effects of COVID-19 on people experiencing homelessness.

## 2 Materials and methods

### 2.1 Design and setting

From mid-2020 until May 2022 we conducted the study “Corona and Homelessness” (financed by ZonMw, i.e., the Netherlands Organisation for Health Research and Development) on the prevalence of COVID-19 infection among people experiencing homelessness in the Netherlands, the organization of COVID-19 preventive measures among them, and the impact of the pandemic on the health and lives of people experiencing homelessness.

As part of this study, three consecutive rounds of semistructured interviews with people experiencing homelessness were performed including, respectively, 67, 55 and 53 persons. The first round of interviews took place in May and June 2020 (after 3 months of the pandemic); the second round took place between December 2020 and February 2021 (after 1 year of the pandemic) and the last round took place in October and November 2021 (after 1.5 years of the pandemic).

### 2.2 Study population

Respondents were recruited through purposive sampling in 7 different Dutch cities: the 5 largest cities Amsterdam, Rotterdam, Den Haag, Utrecht and Eindhoven as well as 2 smaller cities in the south and east of the Netherlands (Nijmegen and Tilburg). The largest Dutch city, Amsterdam was included in the first round, but could not be included in the last two rounds of interviews due to staff shortages and other problems related to the COVID-19 infection wave at that time. We included the data from Amsterdam in this paper.

For the recruitment of people experiencing homelessness, professionals working in shelters, trusted intermediaries, and experts through experience approached people in homeless shelters and explained the study purpose and methods. Diversity was sought in age, sex, country of origin, and geographical location. After receiving informed consent, the people experiencing homelessness were interviewed face-to-face by one of the researchers. In the first round of interviews, some of the interviews were conducted by phone due to the lockdown, and not all shelter locations allowed researchers to interview people experiencing homelessness on-site.

### 2.3 Data collection and analysis

The topic list for the first round of semistructured interviews was developed based on the literature on effects of preventive measures and of epidemics on the lives and health of people who experience homelessness, and the personal experiences of the research team and advisory board of the study that included experts through experience with homelessness, doctors and nurses providing care for people experiencing homelessness, professionals working in shelter homes and representatives of municipalities. The topic list contained questions on characteristics of the participant (age, gender, country of birth, years of homelessness, sleeping place) and on their experienced mental and physical health. These questions were repeated in the second and third round of interviews, to get insight in changes in experienced health during the pandemic.

In addition, each round of interviews included questions on specific topics that were relevant at that moment in time. As such, the focus of the first round of interviews was on compliance with the COVID-19 public health measures. The second round focused on the changes in care and shelter for people experiencing homelessness, the vaccination strategy, and the impact of the pandemic on their everyday lives. The third round specifically focused on the impact of the advancing pandemic and the implications for their future prospects.

All interviews were audio-recorded, transcribed, and processed with the soft-ware program Atlas.ti version 8. As we wanted to report experiences, meanings and the reality of participants, an inductive thematic analysis approach was applied as a realist method (22). We started with inductive coding, conducted by the authors JS and TL, independently of each other. In each round at least 5 interviews were also coded by MM. Codes were compared and differences were discussed by JS and TL until an agreement was reached. In the event of disagreement, a third researcher (MM) was consulted. The next step was to collating these codes into themes and subthemes. Some which were explicitly asked about in the interview like “mental health” and others found as patterns in the interviews like “decreased livelihood security.” All the themes were reviewed, discussed and agreed upon by the research team (JS, TL, MM).

Data were first analyzed immediately after the conclusion of each round of interviews and resulted in an interim report. At the end of the third round of interviews, the data of all interviews of all rounds were reviewed and again thematically analyzed resulting in themes that reflected developments during the pandemic like “decreasing physical health.”

### 2.4 Ethical considerations

Ethical approval was obtained from the Medical Ethical Committee of the Radboud University Medical Center (CMO Radboudumc) (CMO) Arnhem-Nijmegen (nr 2020 6428).

## 3 Results

### 3.1 Characteristics of respondents

In the first round 67 people were interviewed, in the second round 55 and in the third round 53. An effort was made to reapproach and interview as many of the same people as possible each round. However, this only succeeded to a limited degree. People experiencing homelessness were either no longer visiting the shelter, could not be reached at the phone number they provided the previous round, or no longer felt the need to participate. Therefore, each round was supplemented with new respondents. Table 1 summarizes the characteristics of the respondents by round and compares them with the homeless population in the Netherlands (5).

**TABLE 1:** Summary of the characteristics of the respondents by round.

	Round 1 (*n* = 67)	Round 2 (*n* = 55)	Round 3 (*n* = 53)	Homeless in Netherlands (2021)
Average age (range)	44 (18–77)	48 (19–76)	44 (20–78)	45 (18–77)
Duration of homelessness	4.3 years (3 weeks–41 years)	2 years (2 days–10 years)	4 years (7 weeks–37 years)	
**Migration background (%)**				
Dutch	43 (64.2%)	33 (60.0%)	32 (60.4%)	40%
EU-country	3 (4.4%)	6 (10.9%)	2 (3.8%)	10%
Non-EU country	21 (31.3%)	16 (29.1%)	19 (35.8%)	50%
**Gender**				
Man	82%	85%	81%	84%
Woman	18%	15%	19%	16%
(Still) homeless due to COVID	–	17%	26%	

### 3.2 Self-perceived mental health status

In general, the self-perceived mental health of respondents deteriorated during the pandemic. In the first round of interviews, 47 out of the 67 (over two third) respondents reported feeling mentally healthy. In the second and third rounds, this number dropped to 32 out of 55 participants (a bit more than half) and 27 out of 53 participants (just half of), respectively. In the first round, which took place in the first 6 months of the pandemic, anxiety was particularly mentioned. Fear of the virus itself was cited as the main cause of increased anxiety.

*“In the beginning I was also really scared because I didn’t know what was happening. Suddenly the virus and you’re not allowed to go outside. I was very scared. I remember calling my dad crying daddy, are we all going to die? No, of course not. Yes. I did feel really awful for a long time” (20-year-old female).*

The decreased mental wellbeing resulted from the accumulation of difficult circumstances, including the feeling of not being able to adequately protect oneself against the virus due to a lack of or expensive protective measures such as face masks, or because it was not possible to adhere to the Public Health and Safety Measures (PHSM) in crowded shelters.

*“Yes, when you’re homeless, you’re already in a less fortunate situation; you don’t have a roof over your head. You’re already stressed because, well, you’re living on the streets. That’s obviously not pleasant, and if you then add COVID-19 to it, well, it’s only more, even worse. (…) Can you imagine? You don’t even have the money to buy a face mask”–(44-year-old man).*

Later in the pandemic, fear subsided but other negative emotions like general distress, gloom, and loneliness became more prevalent.

*“Yes, you become familiar with it at some point. You’re going through it after all, we’re all going through it. So, that fear gradually diminishes” (28-year-old man).*

According to the respondents, feelings of distress were mainly caused by the diminished prospects for a good future in terms of income and outflow to housing. In the last two rounds of interviews, people also reported being bored more often because day programs closed and many people had lost their jobs. This fueled thoughts of gloom and loneliness.

*“Yes, I have felt very lonely. Even though there are people here, I […] Yes, boredom also causes you to get these depressing thoughts and so on” (20-year-old woman).*

For labor migrants and illegal immigrants, stress was also increased by the uncertainty about their possibility to stay indoors during the night. These people were entitled to stay in shelters during lockdown periods, but had to vacate those locations once the lockdown periods were over. This created confusion and stress for many non-entitled people experiencing homelessness.

### 3.3 Factors negatively affecting the lives of homeless individuals during the pandemic

In addition to the fear and distress mentioned above some other specific issues appeared to be related to the mental health of people experiencing homelessness.

#### 3.3.1 Worsening physical health

In the first two rounds of interviews, nearly three quarter of respondents (50 out of 67 and 40 out of 55, respectively) reported feeling (very) healthy physically. In the last round, only slightly more than half of the respondents (27 out of 53) indicated that they felt physically healthy. Participants reported that the change in their perceived physical health was not so much caused by the COVID-19 virus itself but rather by the effects of the introduced PHSM such as the advice to stay indoors as much as possible and to the longer waiting times for and postponement of care due to the pandemic.

*“I do think worse because normally you walk more, go outside more. Now you’re actually, well, my fitness has declined” (32-year-old woman).*

Besides, respondents explained that the pandemic had made them more aware of health in general, resulting in a more critical assessment of their own physical and mental health.

#### 3.3.2 Less accessibility to social and medical care

Respondents in all three interview rounds indicated that the accessibility of agencies, such as the municipality, social services, debt counseling and legal aid, was limited and that contact with social workers had changed. In many cases, contacts could only take place online. However, digital contact proved challenging. Not all people experiencing homelessness had a computer/phone or internet at their disposal, or they were or felt not digitally literate enough. Likewise, respondents found that there was less assistance available, longer waiting times, and that cases were left pending in the dark.

*“The accessibility and approachability is less, also because of infection risk; it’s mostly by phone and then you can’t really look the care provider straight in the eyes and the care provider can’t really get the patient straight in the eyes either. On that point, I think it’s less” (47-year-old man).*

Respondents indicated that the accessibility of agencies was also limited because they had to make an appointment by phone or internet for organizations they previously could access without making an appointment. This decreased the approachability, which seemed to be an important factor for respondents to seek help. Luckily, shelters could still be accessed without appointment, which benefited help-seeking.

Also the accessibility of primary care remained satisfactory: three-quarters of respondents reported being able to visit regularly their primary care physician and/or street doctor who remained easy to reach during the whole pandemic. However, this was not the case for mental healthcare.

#### 3.3.3 Decreased livelihood security: income and housing

The COVID-19 pandemic appeared to be the cause of becoming homeless in one in every 6 respondents in the second round of interviews and even of a quarter of all respondents in the third round. These individuals had either lost their jobs or, in cases where they already did not have a home of their own, were no longer able to stay with family or friends.

Across all three rounds of interviews, respondents mentioned a decrease in income. Some respondents lost their employment due to the pandemic, which had a negative impact on their finances. The COVID-19 pandemic made it more challenging to find work as there were fewer jobs available and there was a general shift toward remote work which was not feasible for people without homes. Besides, income from begging also declined due to store closures and curfews. The uncertainty about the future exacerbated concerns about financial stability.

*“No I did notice that […] because of the corona supply and demand for certain work is not stable and then if it’s not busy and you don’t have a permanent contract yet then the job stops and then you’re without a job again. Then you look online for a job and then you see a lot of working from home full-time” (31-year old woman).*

There was a widespread perception that the COVID-19 pandemic had slowed down the process of realizing housing for people experiencing homelessness. Waiting times were longer, there were fewer available houses, and it was more challenging to obtain the necessary paperwork. All this influenced peoples’ mental state negatively.

*[response to why no longer happy] “Yes, I do laugh occasionally because, you know, when you work in the hospitality industry, you can’t just appear gloomy in front of the guests, but the situation you’re in is completely different. And the prospects of getting a house, well, it’s not that easy, because there are women here who have been here for 2 years or even longer. And I’ve only been here since last year, so yeah” (33-year-old woman).*

#### 3.3.4 Reduced trust in government and agencies

Throughout all three rounds of interviews, a group of respondents expressed an increasing sense of distrust toward authorities, including the government, healthcare system, and legal system. In the first round of interviews, this was particularly evident in their doubts about the origin of the virus and the effectiveness of PHSM. In the latter two rounds, respondents also expressed doubts about the COVID-19 vaccine. Reduced financial security due to the pandemic further contributed to this sense of distrust. Overall, this growing distrust made people feel uneasy and fearful.

Several respondents perceived inconsistencies between the PHSM and the information provided about the coronavirus.

*“Well with the measures. The festival at Zandvoort is allowed to be celebrated lavishly but the hospitality industry is not allowed to open. If you want to go for a beer you need a piece of paper. We do not live in a police state here, but it is slowly beginning to look like one. The more measures are imposed, the more you have to obey, the more scared everyone is” (70-year-old man).*

*“One minute they say this and the next minute they say that. So not credible it came across” (36-year-old woman).*

According to most respondents in all three rounds, the government’s communication about the coronavirus was unclear and ineffective. The information provided, including that shared during press conferences, was often too complex or inconsistent, leading to confusion and anxiety. As a result, it was unclear what was happening or what was expected of individuals, exacerbating feelings of uncertainty and fear and increasing distrust.

*“Communication to the people in a clear way that everyone understands say. Not everyone understands those terms, or political plan. So then more approachable information” (28-year-old man).*

*“You know, because I don’t always understand that either. I don’t understand everything they say, you know, or you have to be really highly educated or something like that to understand everything, yeah, I don’t understand everything” (28-year-old man).*

Some of the respondents had already lost trust in the government and agencies prior to the pandemic due to the inadequate support they experienced in the past. As a result of the COVID-19 crisis, this distrust sometimes led to a reluctance to seek care.

*“[About the government] Now that we’re a year and a half on, I’m also like, yeah look what do I care. When I needed it then you guys weren’t there, so I had to do it myself”(29-year-old man).*

#### 3.3.5 Lack of social contacts and increased irritability due to being locked up

The pandemic had a notable impact on respondents’ social lives. The feelings of loneliness increased. Travel restrictions and fear of contagion made it difficult for them to see family and friends, while their interactions with others on the street were also reduced. Some respondents reported feeling ostracized in public spaces and experiencing more social isolation as a result.

*“Everyone looks at you scared when you say something. I don’t like that” (20-year-old man).*

The closure of the day care center also reduced social contacts.

*“It’s hard to run into each other because there is no more day care” (43-year-old man).*

The result was a feeling of loneliness and very much missing social contacts.

*“You can’t go in anywhere anymore and the sociability is also gone and that’s the most important thing for people” (70-year-old man).*

In the latest round of interviews, some respondents mentioned that coresidents in the shelter were also a significant source of mental distress. Feelings of frustration and irritation toward fellow residents were commonly mentioned, resulting in a feeling of insecurity. According to the respondents, a negative atmosphere prevailed in the shelter that was not beneficial to their own mental wellbeing.

*Yes, just with ups and downs here, because it can be difficult at times, mental health, look, everyone is also here with problems. So you’re already not in a healthy situation actually here. I have my own things to deal with, so for a while I can’t be the person who has to be there for everyone here. You’re not always comfortable in your own skin and that’s hard too. “You don’t only have to deal with people experiencing homelessness here, you also have to deal with addicts, people with criminal backgrounds, you name it. That’s kind of hard to always be mentally healthy then” (28-year-old man).*

### 3.4 Factors positively affecting the lives of people experiencing homelessness during the pandemic

In addition to factors that negatively affected the mental health of respondents, some positive factors were mentioned. In all three rounds of interviews, respondents mentioned that they considered it very pleasant that shelters were now open for 24 h with fewer people in each dormitory. Having a place to stay during the day gave many respondents much-needed peace of mind. Additionally, the smaller dormitories, aimed at reducing the risk of infection, were felt as beneficial. Respondents reported getting a better night’s sleep.

*“Well and thankfully it’s a lot calmer because there may be fewer people” (37-year-old man).*

For respondents, this much-needed peace of mind created more space in their minds to think about and plan their future. Many people experiencing homelessness felt more tranquility due to fewer people around them or fewer activities.

*“You also get to know yourself very well. I’ve noticed that too. The bad and the good sides. I think I’ve become somewhat stronger during this period, faced more challenges. I’ve been thinking more about life and about what I want”–20-year-old woman.*

## 4 Discussion

### 4.1 Main findings

During the COVID-19 pandemic, homeless individuals in the Netherlands experienced a sharp deterioration of their mental as well as physical health. The negative impact on mental health was largely attributed to the collateral consequences of the pandemic and the preventive public health measures, such as loss of income, reduced social interactions, limited access to social and medical services, and increased feelings of distrust toward agencies. However, the emergence of small-scale shelters was identified as a positive aspect of the pandemic, providing individuals with a peaceful and reflective space to contemplate and plan for their future.

Also studies in other countries mentioned the negative impact of the COVID-19 pandemic on the mental health of people experiencing homelessness (16, 23–25). Verheul et al. (4) showed that mental distress was already before the start of the pandemic highly prevalent among persons experiencing homelessness in the Netherlands (4). Our study adds to this body of knowledge insight into to causes of this deterioration, highlighting the collateral consequences of the pandemic and public health measures, such as loss of income, a heightened sense of hopelessness and reduced access to social and medical services. Our findings underscore the importance of considering social and economic factors when thinking about the impact of pandemics on socially vulnerable populations.

In line with the our study, others also found that the pandemic worsened the already precarious situation of people experiencing homelessness and further diminished their already limited resources (26, 27), reduced the accessibility of healthcare and social support especially in persons with multiple challenges like mental health issues or substance abuse or who resided illegally in the Netherlands, resulting in higher risks of being excluded from social and state safety nets, thereby exacerbating their marginalization. Like our study, the study among domestic workers who reside illegally in the Netherlands also found that preventive measures had a negative impact on the mental health and wellbeing of participants, with fear of contracting the virus, social isolation, and limited access to healthcare cited as significant sources of stress and anxiety. All these findings highlight the importance of considering the unique challenges faced by marginalized populations during times of crisis and the need for targeted support programs to address these challenges. They emphasize the urgent need for interventions that prioritize the mental health and wellbeing of vulnerable populations, including people experiencing homelessness, during the COVID-19 pandemic and beyond.

In line with our findings also other studies revealed positive impacts of the pandemic besides the negative ones mentioned above, like more tranquility and mental space when 24/7 access to shelters is realized, with a limited number of persons per dormitory (28). There is an urgent need to learn from these often unexpected, positive impacts and see how they can enhance future pandemic responses as well as improve care and support for homeless individuals.

### 4.2 Strengths and limitations

During the COVID-19 pandemic, the national network of street doctors and street nurses (NSG), in conjunction with various shelter locations, provided access to areas where persons experiencing homelessness typically congregate. This facilitated the recruitment of our respondents resulting in the inclusion of a diverse group of people from both large and small cities across the country in all three rounds of interviews. However, it is important to acknowledge the potential for selection bias, as only individuals who visited the shelter locations and were fluent in either Dutch or English were invited to participate, thereby excluding rough sleepers and those who only speak a different language.

### 4.3 Recommendations

This study revealed various factors that are essential for providing effective care and support for people experiencing homelessness during the COVID-19 pandemic. Factors, that are also important beyond this pandemic and result in the following recommendations for practice, policy and future research.

• Access to shelter and to care and support services has to remain open and available at all times.
• All persons, regardless their legal entitlements, should have access to healthcare including regular testing for infections if applicable, as well as to mental healthcare and social support.
• During crises specific attention should be paid to income security of people experiencing homelessness.
• Shelters should be open 24/7 and the number of persons per dormitory should be restricted.
• The effects of such measures on the mental and physical health of persons involved, as well as on their ability to improve their lives (getting housing and jobs) should be studied.
• People experiencing homeless should have access to support services at all times, and to engaging activities that also could ensure social contacts.
• Public health related communication should be clear and consistent in order to minimize anxiety and confusion.

## 5 Conclusion

The COVID-19 pandemic and its preventive public health measures negatively impacted the mental and physical health of people experiencing homelessness. This deterioration was related to the collateral consequences such as loss of income, reduced social interactions and reduced access to social and medical services. Some changes in accessibility and scale of shelters and dormitories were positive.

Future pandemic preparedness plans should acknowledge these negative effects and prevent them as much as possible by ensuring access at all times to shelters, maintaining access to healthcare and support services, realizing clear communication and ensuring income security.

Smaller-scale and 24/7 accessible shelters should be implemented regardless of epidemics, to improve the health of people experiencing homelessness.

By addressing these issues, policymakers and service providers together can ensure that people experiencing homelessness will receive at all times the support they need to navigate through challenging times with dignity and security.

## Data availability statement

The datasets presented in this article are not readily available because there are Ethical restrictions for sharing all of our data publicly. We researched people experiencing homelessness in a few Dutch cities. This is a relative small group in the Netherlands for which it is difficult for the results not to be indirectly traceable. Moreover, this is a vulnerable group which is why we want to be extra careful. Given the delicate information and the easy to person retraceable information, we do not want to disclose the entire dataset. We have placed in the DANS repository all data that can be shared (e.g., summary findings). These can be found at: DOI: 10.17026/dans-2c7-wksz. In addition, the data will be made available upon request. In case of a request, we will have an Ethical Board look into which data can be shared. Requests to access the datasets should be directed to TL, kwaliteitsteam.elg@radboudumc.nl, data stewards ELG.

## Ethics statement

The studies involving humans were approved by the Medical Ethical Committee of the Radboud University Medical Center (CMO Arnhem-Nijmegen). The studies were conducted in accordance with the local legislation and institutional requirements. Written informed consent for participation in this study was provided by the participants’ legal guardians/next of kin. Written informed consent was obtained from the individual(s) for the publication of any potentially identifiable images or data included in this article.

## Author contributions

TL: Conceptualization, Data curation, Formal analysis, Investigation, Methodology, Project administration, Supervision, Validation, Writing – original draft, Writing – review and editing. JS: Formal analysis, Investigation, Software, Writing – review and editing. MM: Conceptualization, Funding acquisition, Supervision, Writing – review and editing.

## References

[1] Chiriboga D Garay J Buss P Madrigal R Rispel L. Health inequity during the COVID-19 pandemic: A cry for ethical global leadership. *Lancet.* (2020) 395:1690–1. 10.1016/S0140-6736(20)31145-4 32419711 PMC7225689

[2] Aldridge R Story A Hwang S Nordentoft M Luchenski S Hartwell G Morbidity and mortality in homeless individuals, prisoners, sex workers, and individuals with substance use disorders in high-income countries: A systematic review and meta-analysis. *Lancet.* (2018) 391:241–50. 10.1016/S0140-6736(17)31869-X 29137869 PMC5803132

[3] Nusselder W Slockers M Krol L Slockers C Looman C van Beeck E. Mortality and life expectancy in homeless men and women in Rotterdam: 2001-2010. *PLoS One.* (2013) 8:e73979. 10.1371/journal.pone.0073979 24098329 PMC3788767

[4] Verheul Msc M Laere I Muijsenbergh D Genugten W. Self-perceived health problems and unmet care needs of homeless people in the Netherlands: The need for pro-active integrated care. *J Soc Interv Theory Pract.* (2020) 29:21–40.

[5] CBS. *Stijging van het aantal daklozen tot stilstand gekomen.* Heerlen: CBS (2021).

[6] Kelly D Murphy H Vadlamudi R Kraut R Dalessio K Malani A Successful public health measures preventing coronavirus disease 2019 (COVID-19) at a Michigan homeless shelter. *Infect Control Hosp Epidemiol.* (2021) 42:1155–6. 10.1017/ice.2020.439 32843114 PMC7503037

[7] World Health Organisation. *Considerations for implementing and adjusting public health and social measures in the context of COVID-19: Interim guidance 2020.* Geneva: World Health Organisation (2020).

[8] Rijksoverheid. *Richtlijn opvang dak en thuisloze mensen [guideline homeless shelter].* The Hague: Rijksoverheid (2020).

[9] Kirby T. Evidence mounts on the disproportionate effect of COVID-19 on ethnic minorities. *Lancet Respir Med.* (2020) 8:547–8. 32401711 10.1016/S2213-2600(20)30228-9 PMC7211498

[10] Okonkwo N Aguwa U Jang M Barré I Page K Sullivan P COVID-19 and the US response: Accelerating health inequities. *BMJ Evid Based Med.* (2020) 26:176–9. 10.1136/bmjebm-2020-111426 PMC7299650 32493833

[11] Williamson E Walker A Bhaskaran K Bacon S Bates C Morton C Factors associated with COVID-19-related death using OpenSAFELY. *Nature.* (2020) 584:430–6. 32640463 10.1038/s41586-020-2521-4 PMC7611074

[12] Kuehn B. Homeless shelters face high COVID-19 risks. *JAMA.* (2020) 323:2240. 10.1001/jama.2020.8854 32515823

[13] Wood L Davies A Khan Z. COVID-19 precautions: Easier said than done when patients are homeless. *Med J Australia.* (2020) 212:384-384.e1. 10.5694/mja2.50571 32266965 PMC7262153

[14] Aitken E. Covid-19: Opportunity to improve crisis responses to homelessness? *J R Coll Physicians Edinb.* (2021) 51(Suppl. 1):53–62. 34185039 10.4997/JRCPE.2021.242

[15] Benfer E Vlahov D Long M Walker-Wells E Pottenger J Gonsalves G Eviction, health inequity, and the spread of COVID-19: Housing policy as a primary pandemic mitigation strategy. *J Urban Health.* (2021) 98:1–12. 10.1007/s11524-020-00502-1 PMC7790520 33415697

[16] Bertram F Heinrich F Fröb D Wulff B Ondruschka B Püschel K Loneliness among Homeless Individuals during the first wave of the COVID-19 pandemic. *Int J Environ Res Public Health.* (2021) 18:3035. 10.3390/ijerph18063035 PMC7999173 33809484

[17] Rodriguez N Lahey A MacNeill J Martinez R Teo N Ruiz Y. Homelessness during COVID-19: Challenges, responses, and lessons learned from homeless service providers in Tippecanoe County, Indiana. *BMC Public Health.* (2021) 21:1657. 10.1186/s12889-021-11687-8 34507565 PMC8432956

[18] Baggett T Keyes H Sporn N Gaeta J. Prevalence of SARS-CoV-2 infection in residents of a large homeless shelter in Boston. *JAMA.* (2020) 323:2191–2. 10.1001/jama.2020.6887 32338732 PMC7186911

[19] Lewer D Braithwaite I Bullock M Eyre M White P Aldridge R COVID-19 among people experiencing homelessness in England: A modelling study. *Lancet Respir Med.* (2020) 8:1181–91. 10.1016/S2213-2600(20)30396-9 32979308 PMC7511167

[20] Van Loenen T Mennis E Hobus M van den Muijsenbergh M. *Daklozen en corona. COVID19 monitor onder dakloze mensen [Homeless and COVID. Factsheet 5. Covid19 Monitor among homeless people].* Nijmegen: Radboudumc (2021).

[21] Richard L Booth R Rayner J Clemens K Forchuk C Shariff S. Testing, infection and complication rates of COVID-19 among people with a recent history of homelessness in Ontario, Canada: A retrospective cohort study. *CMAJ Open.* (2021) 9:E1–9. 10.9778/cmajo.20200287 33436450 PMC7843074

[22] Braun V Clarke V. Using thematic analysis in psychology. *Qual Res Psychol.* (2006) 3:77–101.

[23] Dost K Heinrich F Graf W Brennecke A Kowalski V Leider A Predictors of loneliness among homeless individuals in Germany during the COVID-19 pandemic. *Int J Environ Res Public Health.* (2022) 19:12718. 10.3390/ijerph191912718 36232018 PMC9566392

[24] Rodriguez N Martinez R Ziolkowski R Tolliver C Young H Ruiz Y. “COVID knocked me straight into the dirt”: Perspectives from people experiencing homelessness on the impacts of the COVID-19 pandemic. *BMC Public Health.* (2022) 22:1327. 10.1186/s12889-022-13748-y 35820879 PMC9275174

[25] Scarlett H Davisse-Paturet C Longchamps C Aarbaoui T Allaire C Colleville A Depression during the COVID-19 pandemic amongst residents of homeless shelters in France. *J Affect Disord Rep.* (2021) 6:100243. 10.1016/j.jadr.2021.100243 34632442 PMC8487751

[26] Albon D Soper M Haro A. Potential implications of the COVID-19 pandemic on the homeless population. *Chest.* (2020) 158:477–8. 32389724 10.1016/j.chest.2020.03.057 PMC7205705

[27] van den Muijsenbergh M Torensma M Skowronek N de Lange T Stronks K. Undocumented domestic workers and coronavirus disease 2019: A qualitative study on the impact of preventive measures. *Front Commun.* (2022) 7:736148. 10.3389/fcomm.2022.736148

[28] Maxine M Rachael G Jane A. How COVID-19 has impacted access to healthcare and social resources among individuals experiencing homelessness in Canada: A scoping review. *BMJ Open.* (2022) 12:e058233. 10.1136/bmjopen-2021-058233 35914918 PMC9344594

